# The flexibility of ACE2 in the context of SARS-CoV-2 infection

**DOI:** 10.1016/j.bpj.2020.10.036

**Published:** 2020-11-13

**Authors:** Emilia P. Barros, Lorenzo Casalino, Zied Gaieb, Abigail C. Dommer, Yuzhang Wang, Lucy Fallon, Lauren Raguette, Kellon Belfon, Carlos Simmerling, Rommie E. Amaro

**Affiliations:** 1 Department of Chemistry and Biochemistry, University of California, San Diego, La Jolla, California; 2 Department of Chemistry, Stony Brook University, Stony Brook, New York; 3 Laufer Center for Physical and Quantitative Biology, Stony Brook University, Stony Brook, New York

## Abstract

The coronavirus disease 2019 (COVID-19) pandemic has swept over the world in the past months, causing significant loss of life and consequences to human health. Although numerous drug and vaccine development efforts are underway, there are many outstanding questions on the mechanism of severe acute respiratory syndrome coronavirus 2 (SARS-CoV-2) viral association to angiotensin-converting enzyme 2 (ACE2), its main host receptor, and host cell entry. Structural and biophysical studies indicate some degree of flexibility in the viral extracellular spike glycoprotein and at the receptor-binding domain (RBD)-receptor interface, suggesting a role in infection. Here, we perform explicitly solvated, all-atom, molecular dynamics simulations of the glycosylated, full-length, membrane-bound ACE2 receptor in both an apo and spike RBD-bound state to probe the intrinsic dynamics of the ACE2 receptor in the context of the cell surface. A large degree of fluctuation in the full-length structure is observed, indicating hinge bending motions at the linker region connecting the head to the transmembrane helix while still not disrupting the ACE2 homodimer or ACE2-RBD interfaces. This flexibility translates into an ensemble of ACE2 homodimer conformations that could sterically accommodate binding of the spike trimer to more than one ACE2 homodimer and suggests a mechanical contribution of the host receptor toward the large spike conformational changes required for cell fusion. This work presents further structural and functional insights into the role of ACE2 in viral infection that can potentially be exploited for the rational design of effective SARS-CoV-2 therapeutics.

## Significance

As the dominant host receptor of SARS-CoV-2, ACE2 has been the subject of extensive structural and antibody design efforts in aims to curtail COVID-19 spread. Here, we perform molecular dynamics simulations of the homodimer ACE2 full-length structure to study the dynamics of this protein in the context of the cellular membrane. The simulations evidence exceptional plasticity in the protein structure due to flexible hinge motions in the head-transmembrane domain linker region and helix mobility in the membrane, resulting in a varied ensemble of conformations distinct from the experimental structures. Our findings suggest a dynamical contribution of ACE2 to the spike glycoprotein shedding required for infection and contribute to the question of stoichiometry of the spike-ACE2 complex.

## Introduction

**Figure 1:**
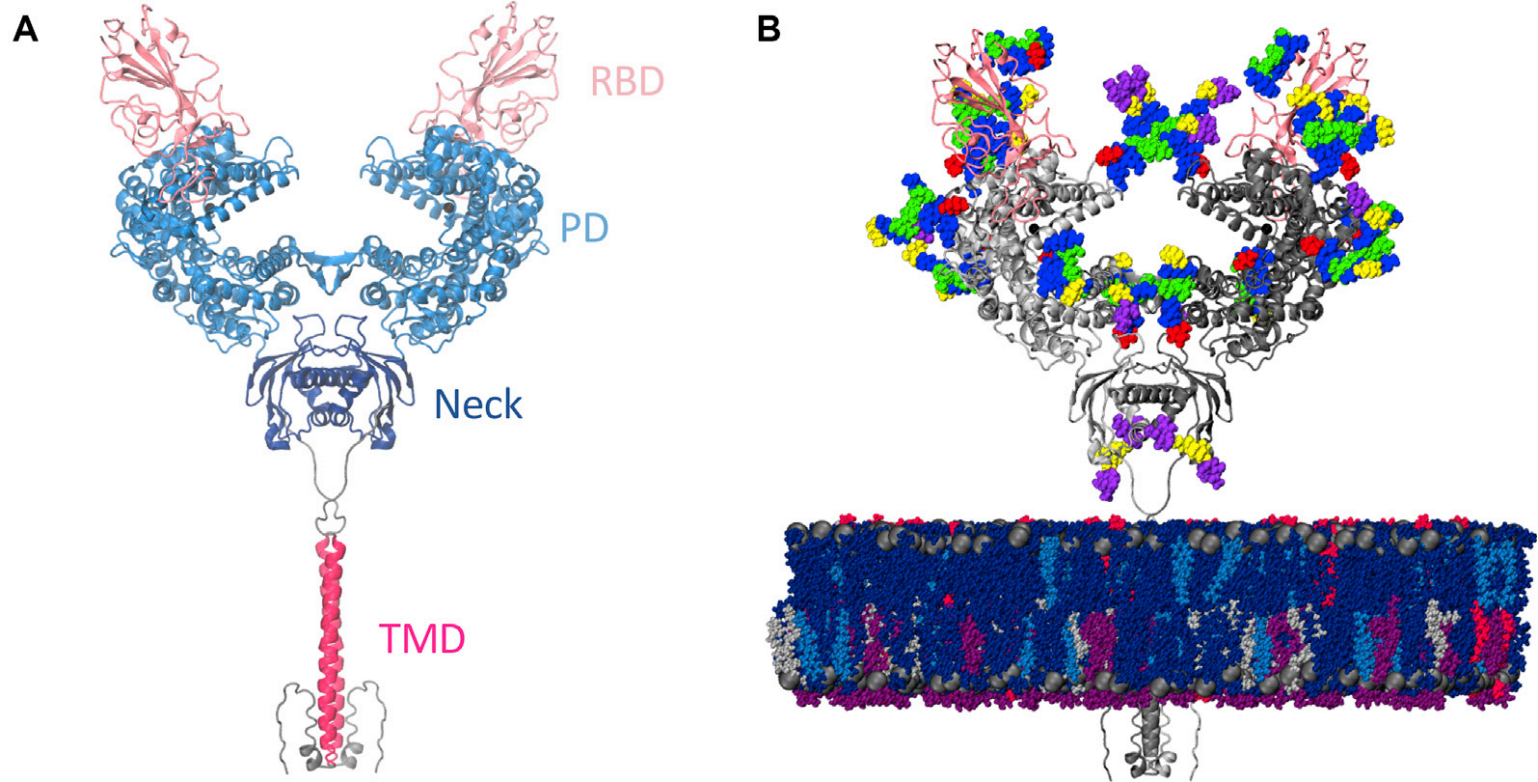
Model structure. (*A*) Full-length ACE2 homodimer protein structure in complex with spike protein RBDs. ACE2 peptidase, neck, and transmembrane domains are shown with cartoons highlighted in blue, navy, and magenta, respectively. Spike RBDs are depicted with pink cartoons. (*B*) Fully glycosylated and membrane-embedded model. ACE2 and RBDs are represented with gray and pink cartoons, respectively. Atoms of N- and O-glycans are shown with per-monosaccharide-colored spheres in which GlcNAc is highlighted in blue, mannose in green, fucose in red, galactose in yellow, and sialic acid in purple. Lipid heads (P atoms) are represented with gray spheres, whereas lipid tails are depicted with a licorice representation using the following color scheme: POPC (*navy*), POPI (*violet*), POPE (*silver*), CHL (*blue*), and PSM (*magenta*). To see this figure in color, go online.

Angiotensin-converting enzyme 2 (ACE2) acts as the extracellular receptor for the severe acute respiratory syndrome coronavirus 2 (SARS-CoV-2) (1, 2, 3), the virus responsible for the COVID-19 pandemic that has catastrophically affected the world since its first identification in December 2019 (4, 5, 6, 7). ACE2 is a membrane protein found in the lungs, kidneys, heart, and intestine cells (8,9) that plays a physiological role in cardiovascular regulation via the cleaving of intermediates in the maturation process of angiotensin, a peptide hormone involved in vasoconstriction control (10, 11, 12, 13, 14). ACE2 is a homodimer with a large claw-like extracellular head domain, a small transmembrane domain, and a short intracellular segment (8). The head can be further subdivided into the catalytic zinc-binding peptidase domain (PD; residues 19–615) (15) and the smaller neck domain (residues 616–726), which is where the majority of the homodimer interactions seems to lie (16). The neck domain is further connected to the single-helix transmembrane (TM) domain by a long linker (Fig. 1 *A*). ACE2 can also function as a membrane-trafficking chaperone for B^0^AT1, an amino acid transporter (17), and it was in fact only in complex with this partner that the single TM helix of ACE2 could be resolved (16).

SARS-CoV (18) (responsible for 8096 cases worldwide in 2002 (19)), and now the closely related SARS-CoV-2, hijack ACE2 as the host cell receptor to its large extracellular spike (S) glycoprotein (1,20). The spike’s receptor-binding domain (RBD) in the “up” conformation binds to ACE2’s PD with high affinity (21), and the resolved ACE2-RBD complex consists of a dimer of heterodimers, with each monomer of the ACE2 homodimer interacting with one RBD (thus forming a heterodimer; Fig. 1 *A*). Cellular recognition and binding to ACE2’s PD via the RBD is proposed to initiate a series of complex conformational transitions in the S homotrimeric protein, leading to the shedding of its S1 subunit and fusion to the host cell membrane driven by the S2 subunit (22, 23, 24, 25, 26), ultimately resulting in the infection of the host cell. Downregulation of ACE2 and accumulation of angiotensin II because of spike binding is also associated with acute respiratory distress syndrome (ARDS) and acute lung failure (27, 28, 29, 30, 31), contributing to SARS-associated symptoms. As such, the S glycoprotein and ACE2-S complex are considered key targets for drug and antibody development efforts aiming to curtail the virus’ remarkable transmissibility and negative effect on human health (1,32, 33, 34, 35), including exploiting the ACE2-S high affinity with recombinant soluble ACE2-antibody constructs (35, 36, 37, 38).

Experimental and biophysical studies of the SARS-CoV-2 RBD and soluble ACE2’s PD complex have suggested structural factors likely responsible for the higher affinity and infectivity of SARS-CoV-2 compared with SARS-CoV (39, 40, 41) and revealed significant dynamics at the RBD-PD interface in the form of rocking motions between the two subunits (41,42). At the individual PD level, opening and closing of the active site cleft has been observed in X-ray structures of the extracellular region (43). Additionally, the recent cryogenic electron microscopy (cryoEM) structures of full-length ACE2 indicate that the homodimer can adopt a less populated open conformation, as defined by the distance between the head domains, in addition to the closed conformation shown in Fig. 1 *A* (16), but little else is known about the global flexibility of this membrane protein. The cryoEM and computational studies of the full-length spike glycoprotein have recently suggested a significant degree of flexibility of the spike’s stalk and at the ACE2-RBD interface (44,45), evidencing the need to study these macromolecular complexes in the context of the cell surface. Here, we perform all-atom molecular dynamics (MD) simulations of the full-length, membrane-embedded, and glycosylated ACE2 homodimer both in the apo state and in complex with RBD to study the dynamics and molecular origins of the ACE2-S flexibility on the host receptor side that evade experimental characterization (46). Seven complex N-glycans and one O-glycan in ACE2 were modeled according to glycoanalytic data (47, 48, 49) as well as glycan N343 in RBD for the RBD-bound simulations (Fig. 1 *B*; Table S1). The B^0^AT1 transporter solved in the cryoEM structure was not included in our simulations to computationally probe the intrinsic dynamics of ACE2. B^0^AT1 is mainly expressed in kidneys and intestines (50), whereas ACE2 can also be found in the lungs and heart tissues, supporting the likelihood that ACE2 can be found uncomplexed with B^0^AT1 upon cellular recognition and binding to S.

The simulations reveal an exceptional structural plasticity of the full-length ACE2 homodimer, pinpointing a large tilting of the head relative to the TM domain as well as profuse mobility of the TM helix in the membrane. Remarkably, the homodimer interface at the level of the neck domains remains stable despite the dramatic motions as well as the ACE2-RBD contacts, emphasizing the high affinity interaction between them. A systematic characterization of glycan-protein and glycan-glycan contacts indicates a possible role of glycan N53 in both homodimer and heterodimer interactions. Overall, the RBD does not seem to significantly affect the dynamics of ACE2 compared with the apo state, although that might differ in the presence of the full-length spike. Taken together, the remarkable ACE2 flexibility indicates a mechanical contribution to the S1 or S2 conformational changes required for cellular fusion and infection and suggests the structural basis for the possibility of finding two or more ACE2 complexes bound to the same S glycoprotein with two or more “RBD-up” conformations.

## Methods

### ACE2 system construction

Coordinates of the ACE2-RBD complex were taken from the full-length cryoEM structure (Protein Data Bank, PDB: 6M17) (16), removing the coordinates from the co-complexed B^0^AT1 dimer. Missing C-terminal residues of the ACE2 transmembrane helices were modeled using I-TASSER (51, 52, 53) based on the known sequence (residues 769–805), whereas missing N-terminal residue coordinates (residues 19–21) were copied from PDB: 6M0J after the alignment of the N-terminal helix. Zinc coordinating residues and a coordinating water molecule were taken from PDB: 1R42 because the zinc coordination site is poorly resolved in PDB: 6M17.

ACE2 and RBD glycosylation was defined according to glycoanalytic data (47,49) and modeled using the Glycan Reader & Modeler tool (54) integrated into Glycan Reader (55) in CHARMM-GUI (56). In total, seven complex, bi-antennary N-glycans and one O-glycan were added to ACE2, as well as 1 N glycan to RBD (Table S1). Only one O-glycan was included at site 730 because analytic data suggest extremely low stoichiometry at the other O-glycosylation sites (48). The apo ACE2 model was created by deleting the RBDs of the complete ACE2-RBD model.

### Membrane modeling

The plasma membrane modeled in this study was composed of 56% POPC, 20% CHL, 11% POPI, 9% POPE, and 4% PSM. The lipid composition was estimated based on the known lipid compositions of mammalian cellular membranes (57,58). It is hypothesized that phospholipids containing charged headgroups such as PI and PS are more likely to face the cytoplasmic side of the membrane and are additionally thought to aid in tolerance of the increased membrane curvature (57). Using a precedent set by a 2014 coarse-grained molecular dynamics study of the asymmetrical mammalian plasma membrane, the lipids were partitioned according to the outer versus inner leaflet enrichment factors of 2.0, 1.2, 0.0, 0.25, and 2.0 for POPC, CHL, POPI, POPE, and PSM, respectively (58). To reduce the chemical complexity of the system for simulation purposes, PS lipids were not included in these calculations. The small percentage (4%) of PS recorded in the literature is represented in the membrane by PI lipids.

An asymmetric 350 × 350 Å lipid bilayer according to the above specifications was generated using CHARMM-GUI’s input generator (56). The lipids were packed to an approximate equilibrium area per lipid of 63 Å^2^. Before insertion of ACE2 and subsequent trimming, the membrane patch contained a total of 2432 POPC, 870 CHL, 460 POPI, 404 POPE, and 128 PSM lipids.

### System preparation and molecular dynamics simulations

Histidine protonation states at pH 7.0 were verified using PROPKA on Maestro (Schrödinger, New York, NY). The models were parametrized using PSFGEN and CHARMM36 all-atom additive force fields for protein, lipids, and glycans (59) fully solvated in TIP3P water boxes (60) with 150 mM NaCl. The total number of atoms is 738,696 for the apo system (size: 18.7 × 18.9 × 23.7 nm) and 783,954 for the RBD-bound system (size: 18.7 × 18.9 × 25.1 nm).

MD simulations were performed on the Frontera computing system at the Texas Advanced Computing Center (TACC) using NAMD 2.14 (61). The systems were first subjected to 10,000 steps of conjugate gradient minimization in which the protein, glycans, lipid heads (P atoms for POPC, POPI, POPE, and POPS and O_3_ atoms for CHL), solvent, and ions were kept fixed. The temperature was subsequentially increased incrementally from 10 to 310 K for 0.5 ns at 1 fs/step for the lipids tails to equilibrate using the NVT ensemble. This was followed by simulation at the NPT ensemble at 1.01325 bar and 310 K for 0.5 ns using 2 fs/step. The systems were then simulated for 2500 minimization steps and an additional 0.5-ns simulation with the protein and glycans harmonically restrained at 5 kcal/mol to allow for environment relaxation. Finally, the systems were further equilibrated for 0.5 ns with all restraints released. Production runs were conducted in triplicates of 1 *μ*s for each system. All simulations were performed using periodic boundary conditions and particle-mesh Ewald (62) with a 12-Å cutoff and a switching distance of 10 Å for evaluation of nonbonded interactions. The SHAKE algorithm (63) was employed to constrain bonds involving hydrogen atoms.

### Analysis

Distributions and average values were calculated considering the total sampling in the three replicas for each system. Except when the dimer structure or interdimer contacts were analyzed, the monomer sampling was accumulated such that the calculations correspond to 6 *μ*s of combined sampling (three replicas × two monomers/replica). Reported uncertainties of average values correspond to the standard deviation across the replicas.

#### ACE2 angles and distances calculations

To quantify the range of motion of ACE2 in the simulations, several angle and distance metrics were developed. Calculation was performed using MDTraj (64) with visualization through VMD (65). The 6M17 cryoEM structure was used as the reference structure.

The head tilt angle relative to the transmembrane domain was calculated by first aligning the dimer’s coordinates to the reference cryoEM TM domains, the angle calculated between the centers of mass of the reference’s dimer PDs (residues 18–600), reference’s TM helices (residues 747–774), and monomer’s PD at each frame in the simulation. The helix tilt angle was computed as the angle between a vector defining the membrane’s normal and a vector connecting residues 741 and 765 at the extremities of the helix.

Revolution angle was calculated between the center of mass of the monomer’s PD in the reference conformation, the center of mass of the reference’s TM domain, and the center of mass of the monomer’s PD at each frame in the simulation after the alignment of the monomer’s TM helices. Buckling angle was calculated using the *xy* projections of the center of mass of monomer’s A PD at frame *f*, the center of mass of the reference dimer’s PDs, and center of mass of monomer’s B PD at frame *f* after alignment of the trajectories to the reference dimer neck domains (residues 617–726).

Distance between the monomer’s head domains in the homodimer was calculated by determining the distance between each monomer’s PD center of mass. Distance between the head domain and membrane corresponds to the minimal distance between the PD’s heavy atoms and membrane’s phosphorous atoms at each frame of the simulation. Distance between TM helices was calculated based on the distance between their centers of mass.

#### Principal component analysis

Principal component analysis was performed using the Scikit-learn library in python 3.6 (66). The monomers in each of the ACE2 homodimer trajectories were saved separately and all aligned to the backbone of the transmembrane helix, residues 747–774. The simulation coordinates of the apo and RBD-bound systems were concatenated and used to fit the transformation function, so that both systems were transformed in the same principal component space.

#### Root mean-square fluctuation and secondary structure analysis

Root mean-square fluctuation (RMSF) of C*α* carbons was calculated using CPPTRAJ (67). Calculations were performed for the head and TM domains separately, after alignment of the backbone atoms of the respective domain. Secondary structure calculation was performed with MDTraj’s compute_dssp function (64) using the simplified three-category assignment scheme. The proportion of frames in which each residue’s secondary structure was assigned as a coil, helix, or strand was computed and compared with the assignment obtained for the starting cryoEM structure.

#### Fraction of native contacts and glycan contacts

Fraction of native contacts was calculated according to Mehdipour and Hummer (68). The 6M17 cryoEM structure was used as the reference structure for identification of native contacts. The ACE2-RBD interface was subdivided into three interacting regions according to the interacting residues pairs listed on Table S2.

A systematic characterization of contacts established by each glycan in the system was performed using MDTraj (64), using a cutoff of 3.5 Å between the heavy atoms.

### S model construction

The spike model was obtained from our previous simulations (69). The simulations included only the solvated spike. All atoms except for the spike protein and glycans were removed, along with the lower part of the stalk region of each protomer (residues 1165–1273). Because the cryoEM model was missing density for portions of the RBD, we replaced the RBD coordinates (residues 355–494 for closed RBD, 339–523 for open RBD) of each protomer in both models with the RBD coordinates from the crystal structure of the RBD bound to ACE2 (PDB: 6M0J (70)). The RBD structure from 6M0J was aligned with the backbone heavy atoms (alpha-carbon, carbonyl-carbon, and nitrogen) of each RBD in the initial spike model. We then grafted the RBD coordinates onto the spike at the hinge region, which resolved missing loops as well as introducing a disulfide bond in the RBD. The remaining disulfides not resolved in the cryoEM structures were assessed based on distance criteria and sequence conservation. The system was built using the *ff14SBonlysc* (71) and GLYCAM (72) force fields for the protein and glycan atoms, respectively. These were explicitly solvated in OPC3 water (73) with a 200 mM NaCl buffer (74). The RBD-up and -down systems both consisted of 1,298,646 atoms, and were simulated on Frontera at TACC, and SDCC at BNL using the pmemd.CUDA module of Amber20. The spike systems were equilibrated using a 10-step protocol. First, the water molecules were minimized for 1000 steps using steepest descent, and then for an additional 9000 steps with conjugate gradient, whereas the rest of the system was positionally restrained with 1 kcal/(mol × Å^2^) restraints. The systems were then heated to 310 K at constant volume, again with all atoms except hydrogens and waters restrained with 100 kcal/(mol × Å^2^) positional restraints. The box size and density were then equilibrated over 1 ns with constant pressure, with the same positional restraints as the previous step. The restraints were then lowered to 10 kcal/(mol × Å) for an additional 1 ns of equilibration, before a second minimization. This minimization consisted of 10,000 steps of conjugate gradient with positional restraints now only applied to backbone atoms (alpha-carbon, carbonyl-carbon, and nitrogen), using a force constant of 10 kcal/(mol × Å^2^). The next three steps of equilibration were MD for 1 ns each at constant NPT with positional restraints on protein backbone atoms at 10, 1, and 0.1 kcal/(mol × Å^2^), respectively. This was followed by a final 1 ns of unrestrained MD at constant NPT before beginning production.

To generate a three-up model of the prefusion spike protein, steered molecular dynamics (SMD) was used as implemented in the Amber NFE toolkit (75). The initial structure was an all-closed model from the equilibration described above. To generate a structure used as reference for steering, the open monomer from the one-up model was aligned to the other two closed monomers by overlapping S2 domains. The closed RBD domains were then replaced with the open model of the aligned monomer. No equilibration was performed on the resulting reference structure because it was not subjected to simulations, and only used as a steering target. Root mean squared deviation (RMSD) was used as the collective variable (CV) to guide SMD. A separate CV was used for the opening of each RBD. In each CV, the RMSD region includes the RBD (residue 338–517) and three helices in S2 (residue 747–782, 946–966, 987–1034). The three RMSDs were simultaneously gradually decreased by SMD from their initial values to 0 during 20 ns of simulation time at 310 K in the NVT ensemble (Fig. S1), with a 4-fs time step enabled by hydrogen mass repartitioning (76), using a spring constant of 10,000 kcal/mol/Å^2^. Final RMSD values were 0.19, 0.19 and 0.20 Å for the three monomers. Weak (1 kcal/mol/Å^2^) positional restraints were applied to the S2 helices, which were relatively stable during SMD simulations.

### Data availability

Additional supporting research data of our models to enable other groups to use and explore this dynamic system in atomic detail (77) may be accessed through the NSF MolSSI and BioExcel COVID-19 Molecular Structure and Therapeutics site at https://covid.molssi.org/.

## Results

### The ACE2 dimer shows pronounced flexibility

**Figure 2:**
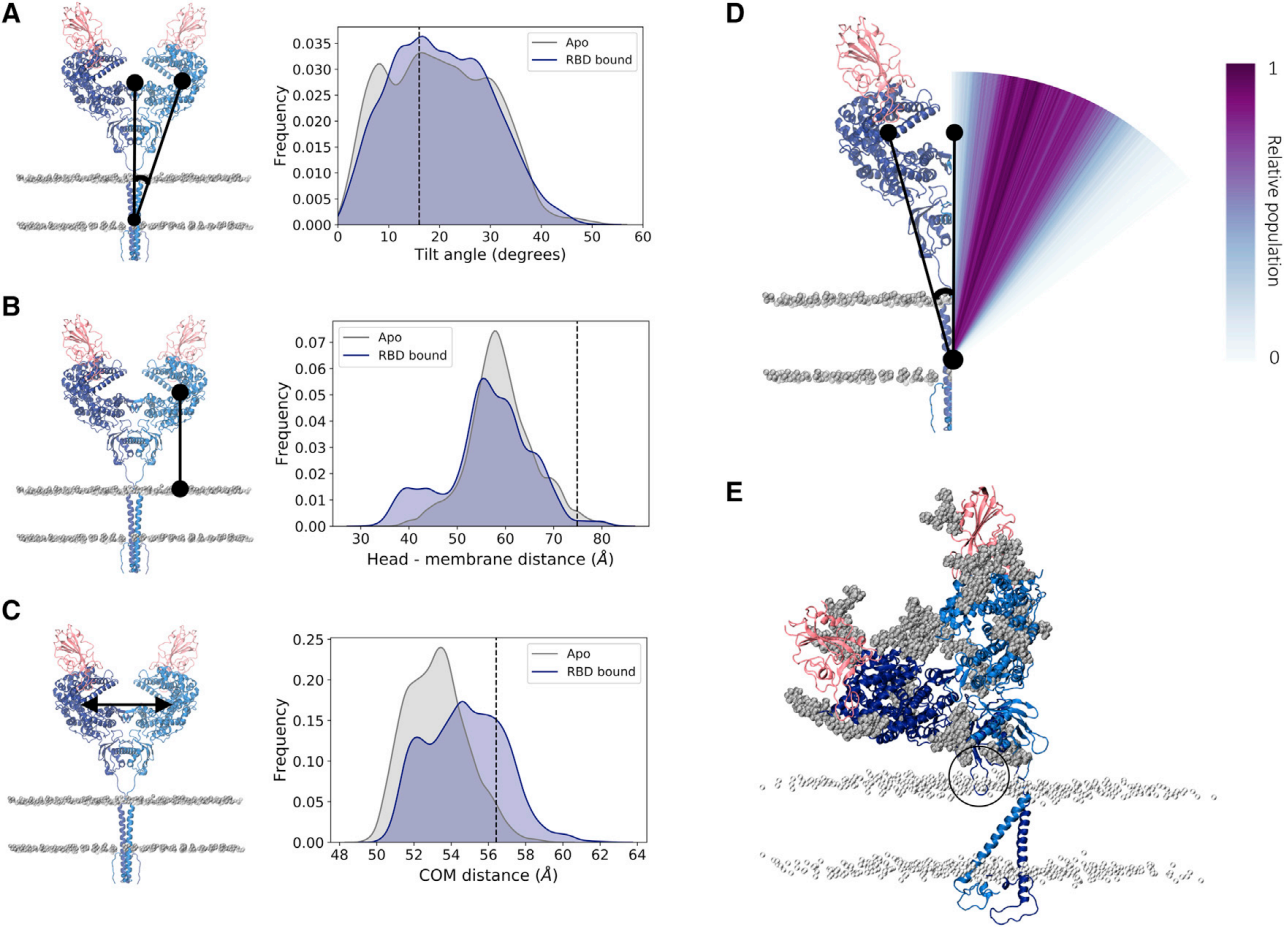
Tilt motion of ACE2. (*A*) Right panel shows head tilt angle distribution relative to the transmembrane domain long axis for apo (*gray*) and RBD-bound (*navy*) simulations, accumulated over the two monomers in the three replicas of each system. The angle value corresponding to the cryoEM conformation is indicated by a black line. Left panel shows a representation of the metric, with ACE2 monomers colored dark and light blue, RBDs colored pink, and phosphorus atoms from membrane’s lipid heads shown in gray in a van der Waals representation. (*B*) Distribution of the minimal distance between peptidase domain (PD) and membrane. (*C*) Distribution of the ACE2 monomer heads’ center of mass distance. (*D*) Visual representation of the tilt angle distribution for the RBD-bound simulations with a color gradient according to the relative population. (*E*) Example of a highly tilted ACE2 homodimer conformation sampled in the simulation. The black circle highlights a membrane-inserted neck domain loop. The ACE2 and RBD glycans are shown in gray, and the membrane’s lipid heads are shown in a silver transparent representation. To see this figure in color, go online.

Simulations of RBD-bound and apo ACE2 evidenced a striking degree of flexibility in the ACE2 homodimer. With respect to the fairly vertical, extended conformation of the initial cryoEM structure (16), the most striking fluctuation observed during the simulations is characterized by a tilt of the head relative to the long axis of the respective monomer’s transmembrane helix. Although each monomer in the reference cryoEM structure displays a tilt angle of 16°, structures in the simulations sample tilt angles that range from 0 to 50° (Fig. 2, *A* and *D*; the distribution is calculated over the combined sampling of the two monomers in the three replicas of each system). This tilt motion, combined with an overall “shrinking” of the initial extended conformation, moves the head toward the membrane, with head-membrane distances varying from 30 to 84 Å and the great majority of conformations (98 and 98.6% of the frames of apo and RBD-bound simulations, respectively) exhibiting the head domain closer to the membrane than the starting cryoEM structure (Fig. 2 *B*). The presence of the RBD does not seem to affect the dynamics, with apo and RBD-bound simulations showing average head-membrane distances of 59.1 ± 6.9 and 56.5 ± 8.8 Å, respectively.

Remarkably, the head tilt motion occurs in a concerted fashion between the monomers, such that as one monomer bends toward the membrane, with large tilt angle values, the other monomer follows this deformation by adopting a more extended conformation with lower angle values (Fig. 2 *E*; Fig. S2). Accordingly, the distance between the head domains fluctuates only slightly, varying by no more than 6 Å from the reference structure (Fig. 2 *C*) and resulting in a stable relative position of the heads within the homodimer. The majority of the conformations display the two heads slightly closer to each other than in the starting cryoEM structure, whereas the presence of the spike RBD shifts the distribution slightly toward more open conformations. In fact, a very small fraction of the conformations access dimer distances equivalent to the head separation observed in the cryoEM open dimer conformation (head center of mass distance of 60 Å (PDB: 6M1D)) (16), but the distribution indicates that this is a rare state.

**Figure 3:**
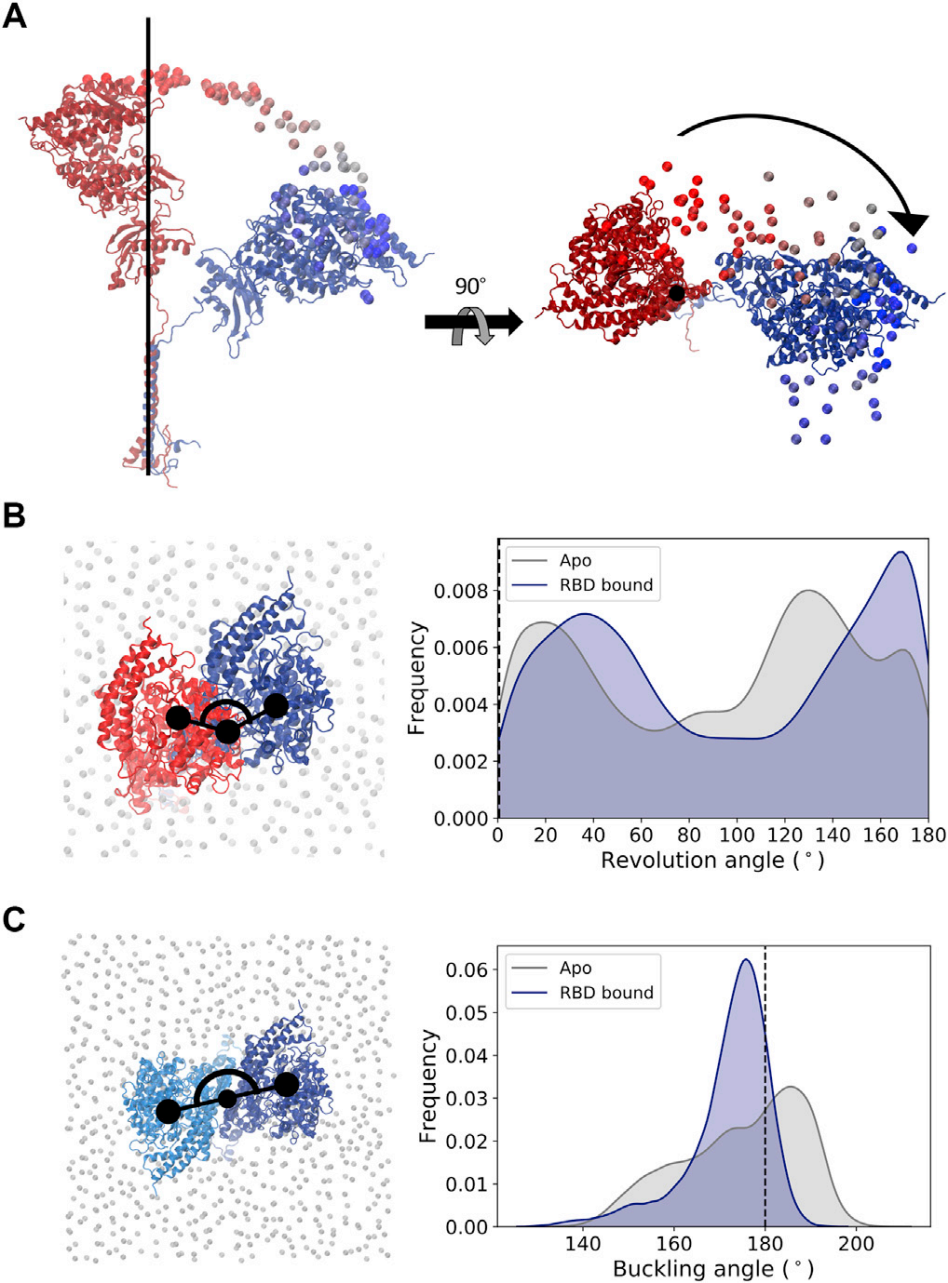
ACE2 revolution relative to a plane perpendicular to the transmembrane helix’s long axis. (*A*) Representation of a monomer’s degree of flexibility in one of the replicas, showing the time evolution of the position of the C*α* atom of Gln325 colored from dark red (t = 0) to dark blue (t = 1000 ns). Conformations aligned to the cryoEM’s reference TM domain C*α* atoms shown in a van der Waals representation and initial and final monomer conformations shown in a cartoon representation. (*B*) Head revolution angle distribution for apo (*gray*) and RBD-bound (*navy*) simulations. The angle value corresponding to the cryoEM conformation (revolution angle = 0) is indicated by a black line. Left panel shows a representation of the metric, with monomer’s head initial position shown in red, the same monomer at a time t in dark blue, and phosphorus atoms from membrane’s lipid heads shown in gray in a van der Waals representation. (*C*) Relative orientation of the monomer’s head in the heterodimer. The ACE2 monomers are colored dark and light blue. To see this figure in color, go online.

**Figure 4:**
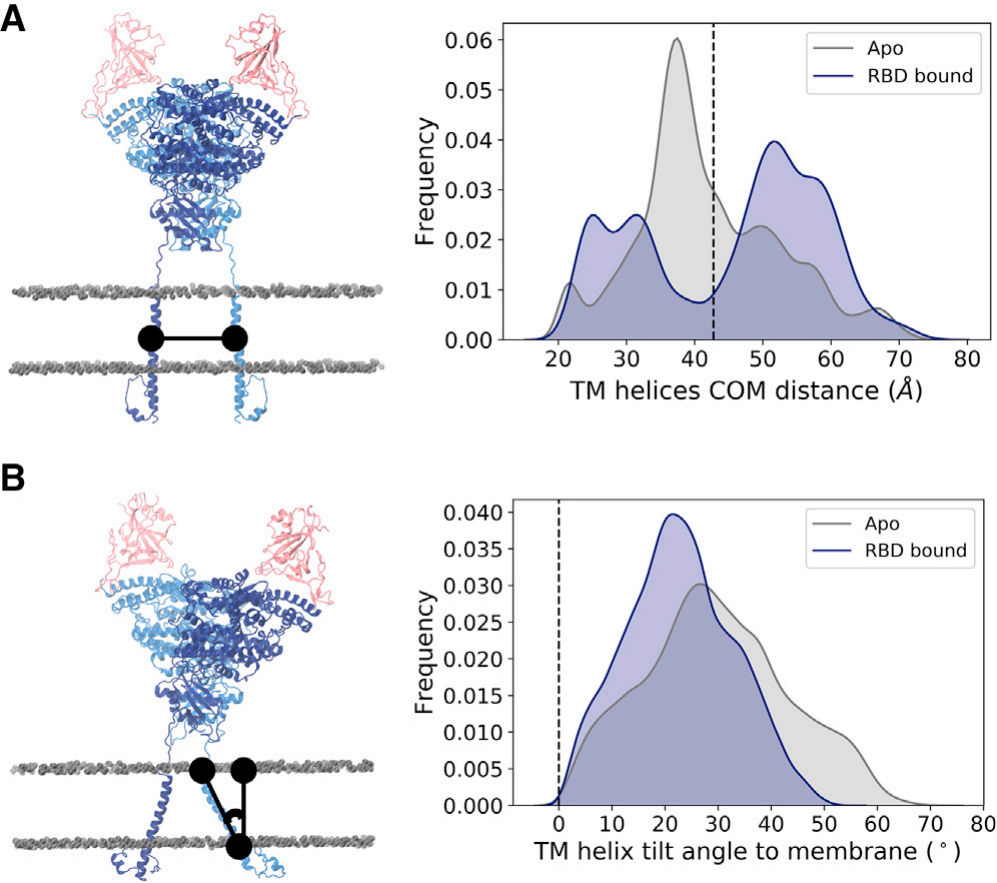
Transmembrane helix dynamics. (*A*) Distance between the center of mass of each monomer’s TM helix. (*B*) TM helix tilt angle relative to the membrane’s normal. The values corresponding to the cryoEM conformation are indicated by a black line. Left panels show representation of the metric, with ACE2 monomers colored dark and light blue, RBDs colored pink, and phosphorus atoms from membrane’s lipid heads shown in gray in a van der Waals representation. To see this figure in color, go online.

In concert with the tilt motion described above, the ACE2 head also undergoes displacement in the *xy* plane around the long axis of the TM helix, as shown for one of the replicas in Fig. 3 *A*. Computation of each monomer’s revolution angle suggests a twisting of the flexible linker that connects the neck to the TM domain, with several significant alternative conformations exhibiting almost 180° rotation of the head from its starting position (Fig. 3 *B*). It is important to highlight that the head revolution is measured here for each monomer independently, after an alignment of that monomer’s transmembrane domain. Because the monomer’s TM helices are not in contact with each other and thus can move independently in the membrane (Fig. 4 *A*), the twisting motion of one of the monomers is not necessarily accompanied by an equivalent twist of the other monomer, avoiding the entanglement of the flexible linkers around each other. Instead, visual observation indicates that the other monomer revolves as a whole around the transmembrane helix of the twisting monomer (Fig. S3), keeping the head dimer interface overall intact. Thus, despite this pronounced motion, the other monomer follows the twist by retaining the heads’ symmetry, and the relative angle between the heads in the *xy* plane remains close to the initial 180° (Fig. 3 *C*).

Principal component analysis of the TM-aligned monomers confirm that the tilt and revolution motions correspond to the greatest variance of the ACE2 head coordinates and emphasize that the head dynamics is a combination of these two main motions: principal components 1 and 2 describe the displacement in two orthogonal planes relative to the TM helix axis and account for 51 and 32%, respectively, of the variance (Fig. S4). The head motion occurs at fast timescales in the order of nanoseconds in both of our apo and RBD-bound simulations, although we expect that this tilting timescale would be longer when considering the full spike bound to ACE2.

Besides the above-described global motions, at a residue level, higher fluctuations are observed for head residues located at the homodimer interface, with particularly high fluctuations at the loop regions Pro336-Val343 and Lys625-Tyr633 (Fig. S5 *A*), which will be discussed in more detail in the following sections. Despite the global motions and backbone fluctuations, the protein secondary structure is overall stable, retaining the motifs identified in the cryoEM structure (Figs. S6 and S7).

The simulations indicate that the conformational variability of ACE2 occurs not only because of flexibility at the linker connecting the transmembrane and head domains but also because of the motions of the transmembrane helix in the membrane. In contrast to other multimeric transmembrane domains, such as the coiled coil trimer of S (21,26,69), each ACE2 monomer is anchored to the membrane by a single helix that does not interact with that of the opposing monomer (Fig. 4 *A*) but rather explores a range of tilt angles relative to the membrane’s normal (Fig. 4 *B*). This tilt motion, however, does not affect the overall integrity of the transmembrane helix, as indicated by RMSF and secondary structure characterization of the TM-aligned conformations (Figs. S5 *B*, S6 *C*, and S7 *C*).

The overall gaussian distributions of the distances and angles measured here emphasize a continuous sampling of the distinct conformations, with no significant energy barriers hindering the transitions. Combined with the multidirectional tilting of each monomer (Fig. S2), the simulations indicate that the deformations occur transiently and with no preferred direction or conformation. Although some distinctions in the geometric descriptor distributions of the apo and RBD-bound simulations can be observed (such as in Fig. 4 *A*), the absence of clear differences and the pronounced protein dynamics do not allow us to exclude the possibility that these differences are simply due to sampling in the simulations and not necessary functionally important. Taken together, our results suggest that the experimentally resolved extended ACE2 structure is likely not a dominant conformation in solution, and the homodimer displays a large ensemble of conformations in the native state.

### ACE2-RBD interface remains stable despite the large ACE2 motions

**Figure 5:**
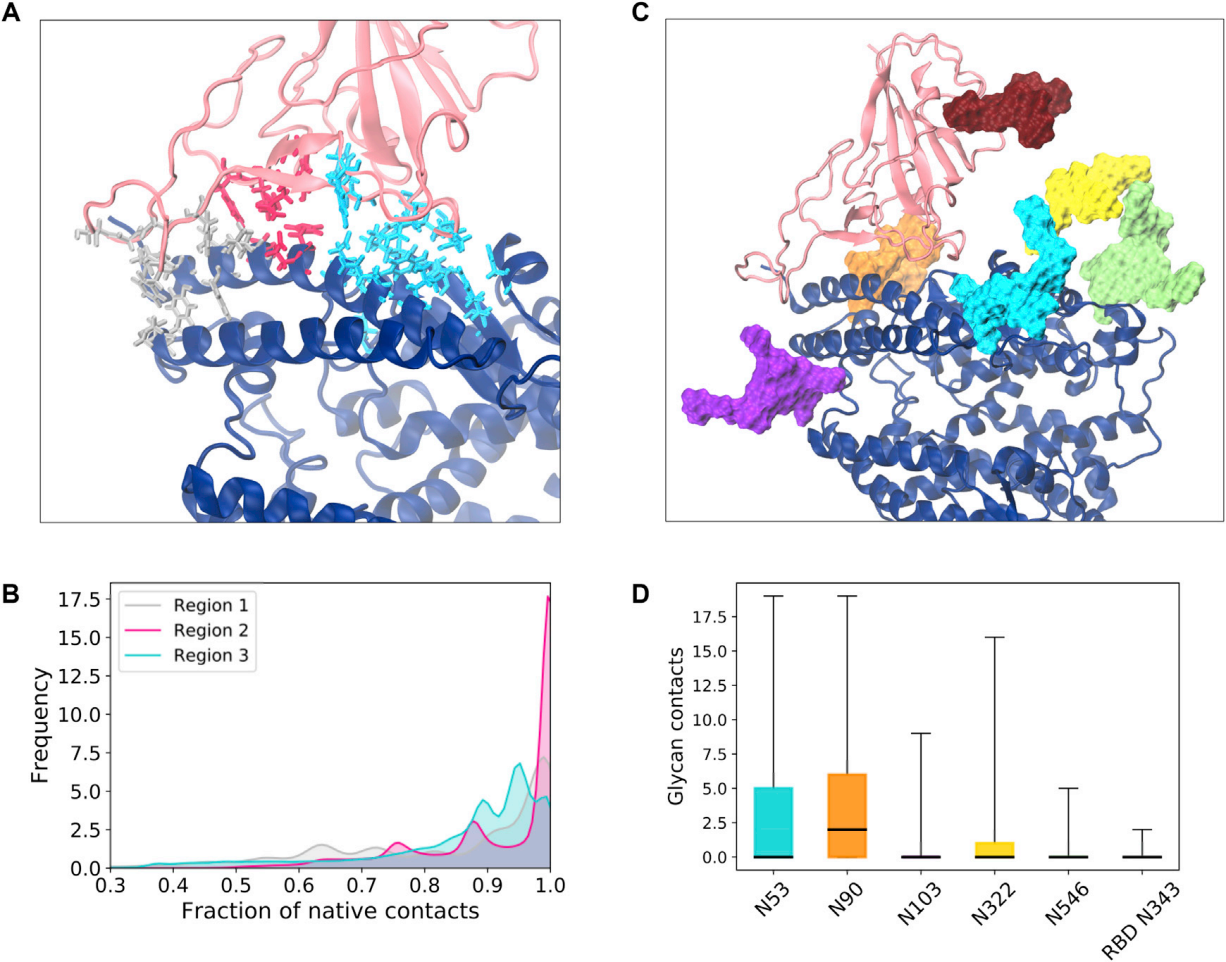
ACE2-RBD interactions. (*A*) Residues in the ACE2-RBD interface colored according to the regions classification (*silver*, *magenta*, and *cyan*) and shown with licorice representation. The ACE2 monomer is shown with dark blue cartoons and the RBD with pink cartoons. Glycans have been omitted from (*A*) for clarity. (*B*) Distribution of the fraction of native contacts in each of the interaction regions. The colors are the same as in (*A*). (*C*) Glycans in the ACE2-RBD interface, shown with surface representation with the following color scheme: N53 (*cyan*), N90 (*orange*), N103 (*purple*), N322 (*yellow*), N546 (*lime*), and N343 (*dark red*). (*D*) Box plot of the number of glycan-protein contacts for the interface glycans shown in (*C*), using the same color scheme. The horizontal black lines indicate the mean value, boxes extend to the lower and upper quartiles, and whiskers show the total range of the data. To see this figure in color, go online.

Despite the dramatic flexibility of the ACE2 dimer, the RBD included in the RBD-bound model retained a large fraction of the native contacts with ACE2 throughout the simulations, with an average fraction of 0.87 ± 0.11 contacts. Although the interface is thus overall stable and the relatively small RBDs accompany the range of motion of ACE2, dividing the RBD-ACE2 interface into three interacting regions (comprising of the two RBD loop regions at the opposite sides of the dimer interface and the central region containing the two short *β*-sheet strands; Fig. 5 *A*; Table S2), we observe that the central region 2 contains the most stable contacts, whereas regions 1 and 3 at the extremities of the interface are less tightly bound, and they sample states with a smaller number of native contacts (Fig. 5 *B*). Analogously, higher backbone fluctuations are observed for the extremities of the RBD binding interface, particularly in the long loop in region 1 (Fig. S5 *C*). Taken together, these observations are in agreement with the rocking motion dynamics at the PD-RBD interface observed in other simulations (41,42).

In addition to protein interactions, five glycans in ACE2 are in close proximity to the RBD and have been suggested to play a role in S binding. In agreement with other studies (48,68), N90, and to a lesser extent N322, of ACE2 establish contacts with RBD. Besides these glycans, we also find that N53 can form a large number of contacts with the RBD residues, whereas the RBD glycan N343 makes very few contacts with ACE2’s head protein residues (Fig. 5 *C*) or glycans (Fig. S8).

### N53 is involved in both homodimer and heterodimer contacts

**Figure 6:**
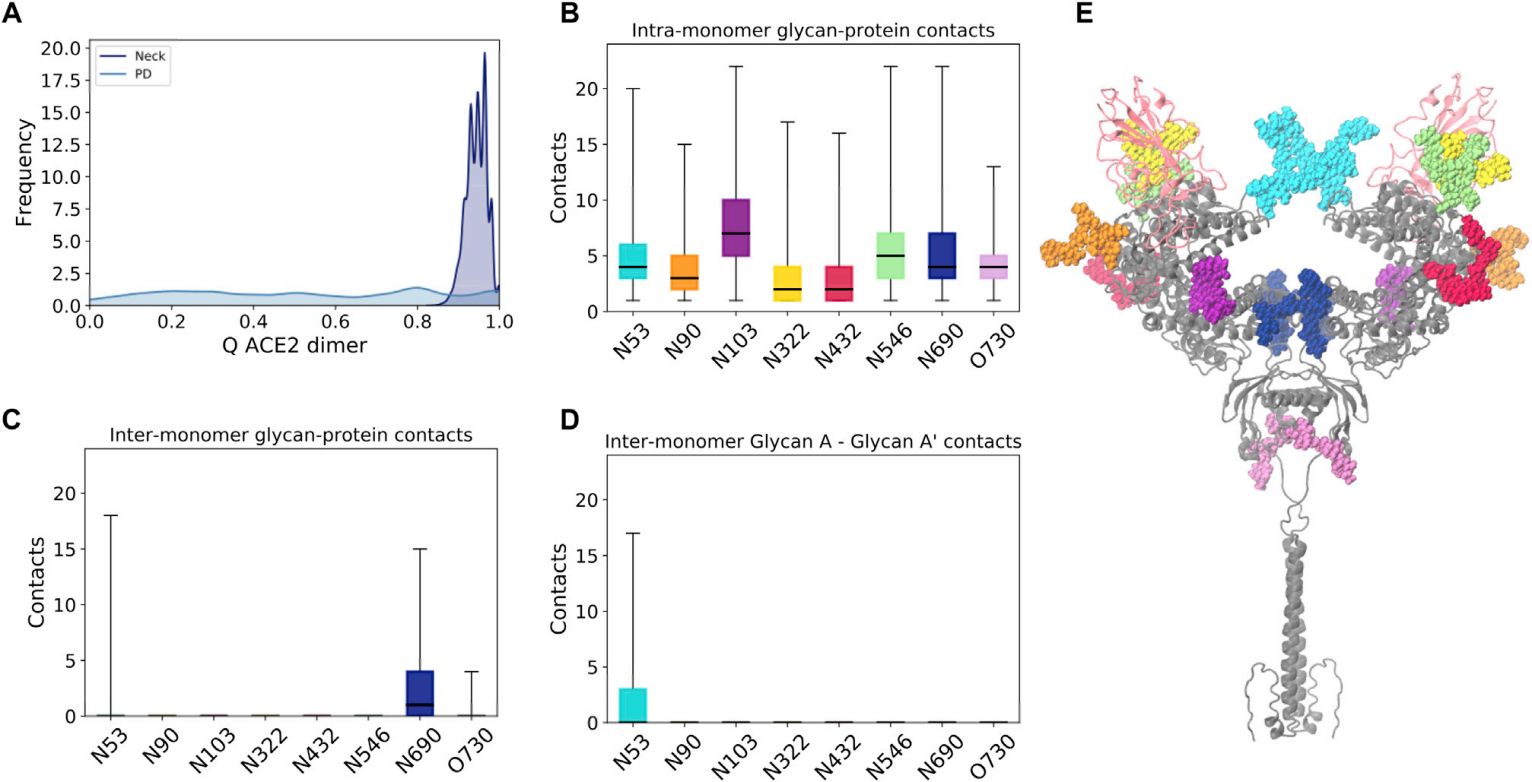
ACE2 homodimer contacts for RBD-bound simulations. (*A*) Fraction of the native contacts between ACE2 monomers considering only protein components of the glycoprotein. The neck and PD interacting regions are depicted separately. (*B*) Total glycan-protein interactions formed within each ACE2 monomer, per glycan. The horizontal black lines indicate mean value, boxes extend to the lower and upper quartiles, and whiskers show the total range of the data. (*C*) Glycan-protein contacts between glycans in one of the monomer and protein residues in the opposite monomer. (*D*) Glycan-glycan contacts between the glycan in one of the monomers (glycan A) and its copy in the other monomer (glycan A’). (*E*) The ACE2 dimer with glycans in a van der Waals representation is colored according to (*B*)–(*D*). The ACE2 protein dimer is colored gray and RBDs in light pink. To see this figure in color, go online.

Besides the ACE2-RBD heterodimer interface, we considered the interactions within ACE2 that could contribute to maintaining such a stable homodimer head interface despite the pronounced flexibility of the protein. Experimental structures and simulations suggest that the majority of the protein contacts in the homodimer are located in the neck domain, with only two other interactions, in the form of hydrogen bonds, that are observed in the larger PD (16,68). In agreement with these observations, we find that the dimer interface is mainly held together in the simulations by residues at the neck (Fig. 6 *A* for RBD-bound simulations and Fig. S9 *A* for apo). Computation of the glycan-protein and glycan-glycan contacts enrich the characterization of the inter- and intramonomer interactions and suggest that although the eight ACE2 glycans form several contacts with protein residues located within the same monomer (Fig. 6 *B*), protein-glycan interactions with the opposite monomer are limited to N53 and N690 (Fig. 6 *C*). Additionally, N53 is the only glycan that can be found to form intermonomer glycan-glycan contacts established between the equivalent N53 copies in each of the monomers (Figs. 6 *D* and S10). In this sense, it is interesting that computational predictions indicate that the disruption of the N53 glycosylation motif because of the mutation at T55, leading to removal of the glycan, can have a destabilizing effect on ACE2 stability (78). Similar to the lack of RBD effect on the ACE2 dimer flexibility, we find that the homodimer contact distributions are comparable between apo and RBD-bound states of ACE2 (Fig. S9).

Our systematic analysis of all glycan interactions thus indicate that N53 is the only glycan involved in both heterodimer (ACE2-RBD; Fig. 5 *C*) and homodimer (intra-ACE2 dimer; Fig. 6 *D*) interactions. The opposite positions of the homodimer and RBD interfaces relative to N53 (Fig. 5 *E*) could suggest a competition for N53 contacts within these dimer interfaces. Indeed, we find that when a large number of N53-RBD contacts are formed, the N53 dimer interface is usually abrogated in the RBD-bound simulations and vice versa (Fig. S11 *B*). However, these interactions are not completely mutually exclusive because N53’ (the N53 glycan in monomer B) can be found involved in both intermonomer and monomer-RBD interactions in replica 1. This glycan’s flexibility is likely the cause of the high backbone fluctuations observed for the neighboring Pro336-Val343 loop region (Fig. S5 *A*) and probably plays a role in conferring a transient nature to the homo- and heterodimer interactions because the N53 homodimer contacts are not consistently formed even in the absence of the competing heterodimer in the apo ACE2 simulations (Fig. S11 *A*). Thus, N53 can still be found highly solvent exposed in the apo state, suggesting optimal conformations for contact with an RBD partner and a role in S binding and infection.

## Discussion

**Figure 7:**
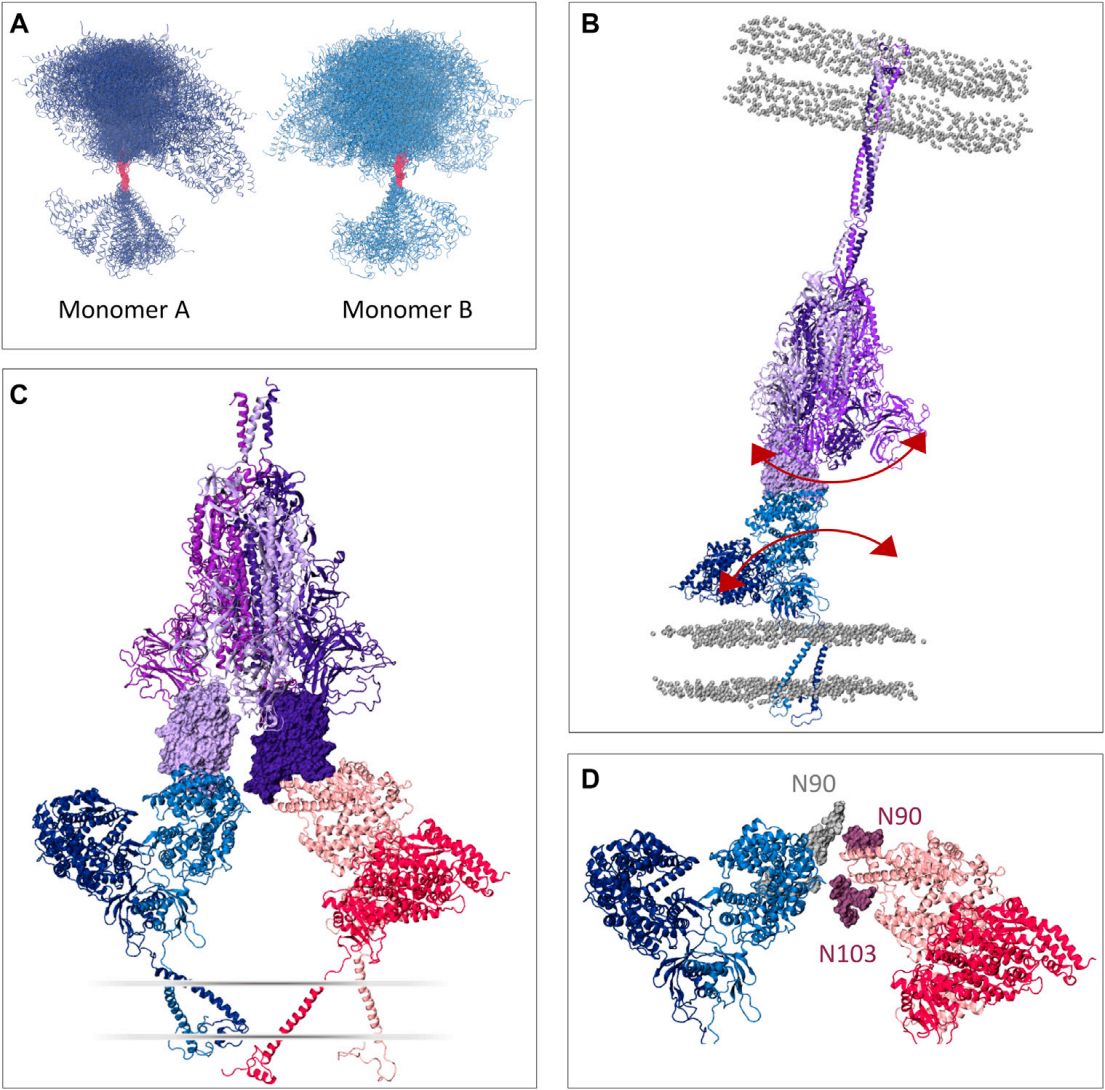
ACE2 flexibility’s impact on S interaction. (*A*) ACE2 monomer conformations taken from equally spaced frames from the simulations, aligned via the flexible linker. (*B*) Proposed effect of ACE2’s flexibility on the spike’s dynamics, communicated through the ACE2-RBD complex. The three chains in the spike model are colored in different shades of purple, with the “up” RBD shown in light purple in surface representation. The phosphorus atoms from membrane’s lipid heads are shown in gray in a van der Waals representation. The red arrows indicate the proposed effect of ACE2 flexibility in the ACE2-S complex dynamics. (*C*) Proposed complex of two ACE2 dimers bound to a single spike, with two RBDs in the “up” conformation. The ACE2 dimers are shown in dark and light blue and dark and light pink, respectively. The RBDs are shown in a surface representation. A schematic of the membrane is indicated. (*D*) Detailed view of the ACE2 heads in (*C*), with N90 and N103 glycans highlighted. To see this figure in color, go online.

All-atom simulations of apo and RBD-bound, full-length, membrane-embedded ACE2 show a striking degree of fluctuation of the homodimer protein, which can be attributed to hinge motions of the large head domain relative to the transmembrane helices, and tilt of the transmembrane helices relative to the membrane’s normal. The head relative motion is due to the flexible linker region connecting the TM helix and the neck domain, whereas the TM helix motion points to a loose anchoring of ACE2 to the membrane. Although the two (head and transmembrane) domains are internally stable, the flexibility of the connecting loop virtually results in a decoupling of these domains’ dynamics (Fig. 7 *A*), leading to sampling of conformations strikingly different than the experimentally observed elongated structure (16). The observed highly tilted conformations position the head domains in the vicinity of the membrane. Although the glycans do not make significant contacts with the membrane lipid heads (with the exception of O730, which is located close to the membrane even in the elongated conformation; Fig. S12), insertion of a loop from the neck domain into the membrane can be observed in the simulations (residues 625–633; Figs. 1 *E* and S13 *A*). Curiously, this loop region exhibits the highest head RMSF values (Fig. S5 *A*). The insertion seems to be driven by hydrophobic contacts involving the side chain of Leu628 and the lipid tails and suggest the existence of lipid-protein interactions that can stabilize the bent conformations (Fig. S13 *B*).

In accordance with our findings, a high deformation propensity was also observed for the TM-neck linker upon normal mode analysis of full-length ACE2 (79). These distinct conformations were likely only observed in the simulations because of the removal of B^0^AT1 from the cocomplexed structure because they seem to bind on opposite sides of the homodimer transmembrane interface and interact with the flexible linker (16). The presence of B^0^AT1 would thus likely reduce the observed ACE2 flexibility, restraining the sampling of highly tilted conformations. However, the expression profiles of ACE2 and B^0^AT1 suggest the likelihood of ACE2 existing in the apo state, especially in lung and heart tissues where B^0^AT1 is not expressed (50). The observed high degree of ACE2 flexibility in the apo form can additionally explain the difficulty in the experimental characterization of the full-length structure.

Remarkably, the conformations of the ACE2 peptidase and neck domains remain stable throughout the simulations, and the homodimer heads retain their relative orientation despite the dramatic global homodimer motions. In a similar fashion, the RBDs included in the holo simulations remained tightly bound to ACE2 throughout the simulations, evidencing the high affinity between them. Glycan-glycan and glycan-protein interactions suggest that the ACE2 homodimer interface is maintained not only via protein interactions at the neck domain but also via intermonomer contacts involving N53 at the top of the PD and N690 closer to the neck. Interestingly, N53 also makes extensive contacts with RBD, suggesting a dual and possibly competing role between homodimer (intra-ACE2) and heterodimer (ACE2-RBD) interactions. This dual nature may be dependent on the length of the N53 glycan, but the glycosylation heterogeneity in ACE2 in general and at this position in particular (49) emphasizes the possibility of intermonomer glycan interactions. Even in the absence of RBD, N53 alternates between being sequestered in homodimer contact and being extended and highly solvent accessible, suggesting a role in RBD binding to this glycan.

Because of the stability of the head domain and RBD interface despite ACE2 body motion, this large flexibility would remain invisible in studies that do not take the full-length character of ACE2 into account, looking, for instance, at only the PD-RBD interactions. However, a recent cryoEM study of S-ACE2 PD complex resolved a continuous swing motion of the ACE2-head-RBD relative to the S trimer body (45). These structural characterizations complement our analysis and suggest how the ACE2 motion would translate in the context of full-length spike. Additionally, it has been found from in situ cryoEM and molecular dynamics simulations that the spike glycoprotein also exhibits conformational plasticity, with hinge motions at three different regions of the stalk trimer (44). Large dynamical variations thus seem to be features of these extracellular glycoproteins. The RBD rocking motion and S conformational variability have been proposed as mechanisms for immune evasion and efficient receptor search in the host cell (44,45), but the similar rocking motion of ACE2 we observed also suggests a mechanical aspect to ACE2-S interaction. The process of S conformational transition upon binding to the receptor and cell fusion remains elusive, but ACE2’s intrinsic flexibility could promote a large swinging motion of the ACE2-S1 complex, providing a mechanical force for the approximation of the two membranes and shedding of S1 toward fusion of the S2 domains into the receptor cell (Fig. 7 *B*).

Finally, one can speculate that the flexibility of the host receptor might allow the accommodation of more than one ACE2 dimer bound to a single S with two or more RBDs in the up conformation. A high efficiency of ACE2 usage was suggested to contribute to SARS-CoV transmissibility (15,80) and thus could be at play for SARS-CoV-2 as well. To investigate this possibility, we extracted a range of ACE2 conformations from the RBD-bound simulations covering different tilt angles and explored the alignment of these structures to a “three RBD-up” spike model. Indeed, we find that two ACE2 dimers can sterically be accommodated by a single spike with interdimer backbone distances no smaller than 10 Å (Fig. 7 *C*). The flexibility of the homodimers could potentially allow for even three ACE2’s per S, opening the possibility of multireceptor usage by the spike glycoprotein for host cell infection. Explicitly considering the glycans in this aligned model evidences that N103 and especially N90 are in close proximity to the neighboring ACE2 dimer (Fig. 7 *D*). Genomic, mutagenesis, and binding studies of ACE2 variants have demonstrated that removal of the N90 glycan due to mutations to the glycosylation motif leads to increased S-ACE2 binding affinity (80, 81, 82), and these observations can thus provide the structural basis for the negative effect of N90 on RBD binding.

## Conclusions

All-atom molecular dynamics simulations of the full-length ACE2 inserted in a mammalian-inspired lipid membrane uncover a significant degree of flexibility of the ACE2 homodimer with consequences for S-ACE2 interaction and SARS-CoV-2 infection and suggest the structural basis for glycan N90’s negative effect on RBD binding. Additionally, we identify the involvement of glycan N53 in ACE2 homodimer and ACE2-RBD heterodimer contacts. Taken together, our findings shed further light onto the mechanisms of viral binding and cell entry required for rational design of effective SARS-CoV-2 therapeutics.

## Author contributions

E.P.B., L.C., and Z.G. built the model of the ACE2 and RBD. A.C.D. built the membrane bilayer. E.P.B. performed the MD simulations and analysis. Y.W. built the three-up RBD, L.F. converted the spike and glycans from Charmm to AMBER and GLYCAM force fields, L.F. performed the MD of the spike structures, and K.B. did the structure grafting. R.E.A. and C.S. designed and oversaw the research project. E.P.B. wrote the article, with contributions from all authors.

## Supporting material

Document S1. Figs. S1–S13 and Tables S1–S2

Document S2. Article plus Supporting Material
