# Supplementary material for: The flexibility of ACE2 in the context of SARS-CoV-2 infection
Source: Biophys J. 2020 Nov 13;120(6):1072–84. doi: 10.1016/j.bpj.2020.10.036 (PMC7661960; doi:10.1016/j.bpj.2020.10.036)
Supplement: Document S1. Figs. S1–S13 and Tables S1–S2 [file mmc1.pdf]

Biophysical Journal, Volume *120*

Supplemental information

The flexibility of ACE2 in the context of SARS-CoV-2 infection

Emilia P. Barros, Lorenzo Casalino, Zied Gaieb, Abigail C. Dommer, Yuzhang Wang, Lucy Fallon, Lauren Raguette, Kellon Belfon, Carlos Simmerling, and Rommie E. Amaro

Supplementary Table 1. Glycan composition for ACE2 and RBD systems.

Protein	Site	Structure
ACE2	N53	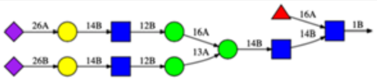
ACE2	N90	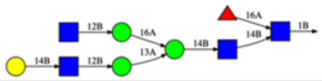
ACE2	N103	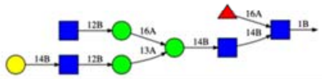
ACE2	N322	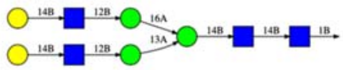
ACE2	N432	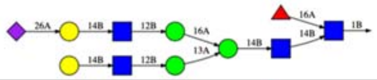
ACE2	N546	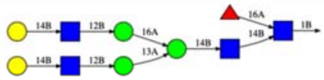
ACE2	N690	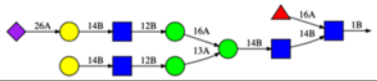
ACE2	O730	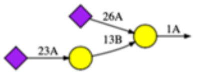
RBD	N343	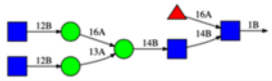

Supplementary Table 2. Composition of ACE2-RBD interface regions.

Region	ACE2 residue	RBD residue
1	GLN24	ALA475
	GLN24	GLY276
	GLN24	SER477
	GLN24	ASN487
	THR27	ALA475
	THR27	TYR489
	PHE28	TYR489
	LYS31	TYR489
	MET82	PHE486
	TYR83	PHE486
	TYR83	ASN487
2	THR27	PHE456
	ASP30	LEY455
	ASP30	PHE456
	LYS31	PHE456
	LYS31	GLN493
	HIS34	TYR453
	HIS34	LEU455
	GLY35	GLN493
3	GLU37	TYR505
	ASP38	TYR449
	ASP38	TYR495
	ASP38	GLY496
	TYR41	GLN498
	TYR41	THR500
	TYR41	ASN501
	GLN42	TYR449
	GLN42	GLN498
	LEU45	GLN498
	ASN330	THR500
	LYS353	GLY496
	LYS353	ASN501
	LYS353	GLY502
	LYS353	TYR505
	GLY354	GLY502
	ASP355	THR500
	ARG357	THR500
	ARG393	TYR505

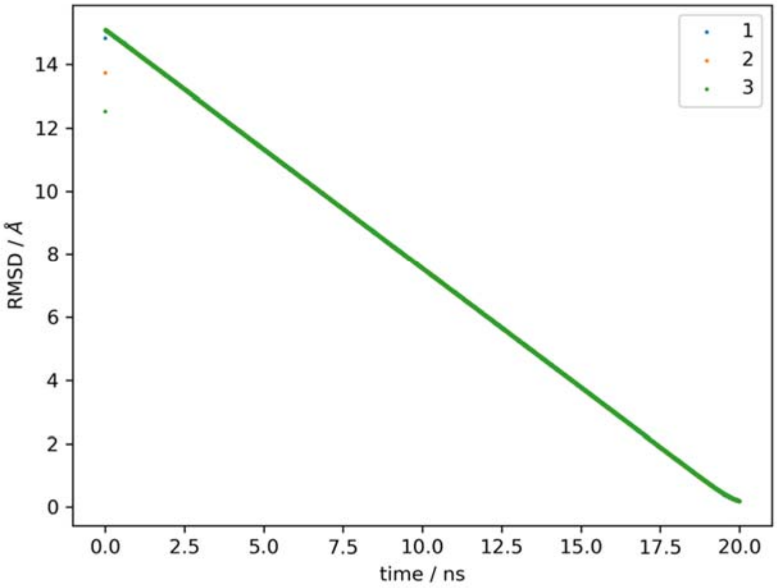

Supplementary Figure 1. RMSD *versus* time for the steered molecular dynamics of the RBD in each spike monomer, relative to the “RBD-up” in the reference structure. The three initial points correspond to the initial values in each monomer.

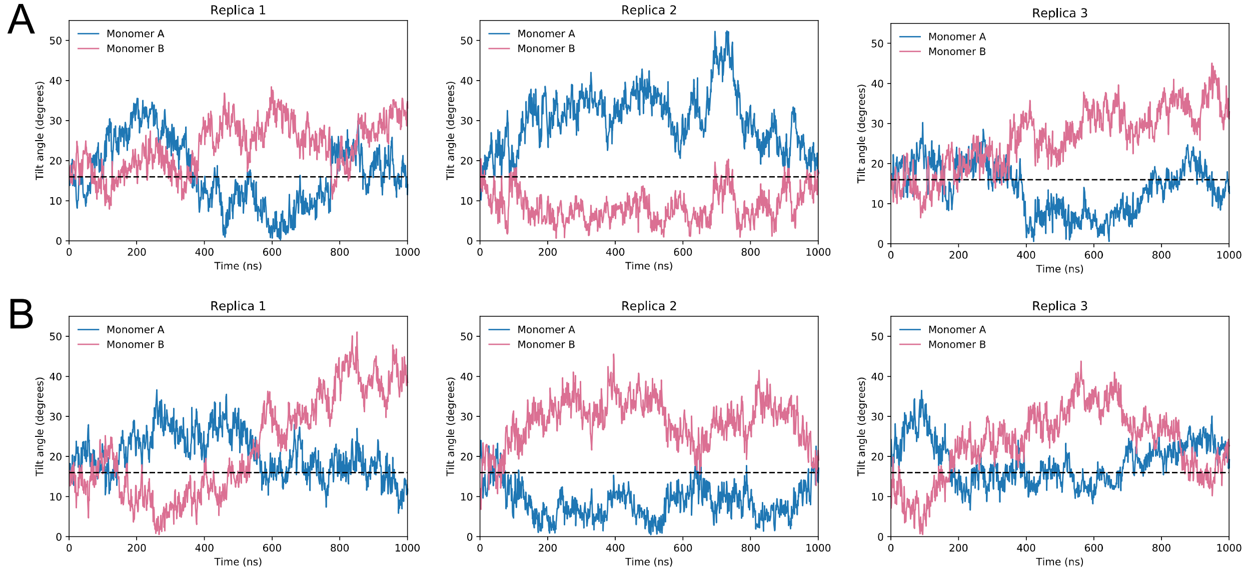

Supplementary Figure 2. Time evolution of the head tilt angle for each monomer in the **(a)** apo and **(b)** RBD-bound simulations. Tilt angle of the reference cryo-EM structure indicated by a black horizontal line.

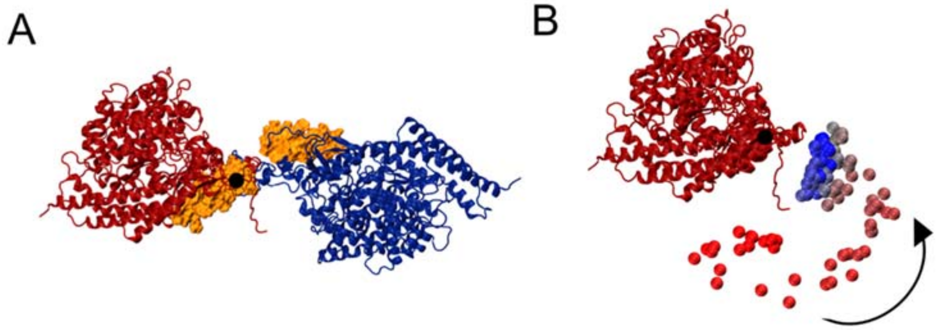

Supplementary Figure 3. (a) Representation of the change in position of the ACE2 homodimer binding interface (in orange surface representation) for the same MD initial and final monomer conformations shown in Figure 2a. **(b)** Displacement of the TM domain of the second monomer following motion of the monomer indicated in (a). Time evolution of the position of the Cα atom of residue 760 in the TM helix colored from dark red (t=0) to dark blue (t=1000 ns).

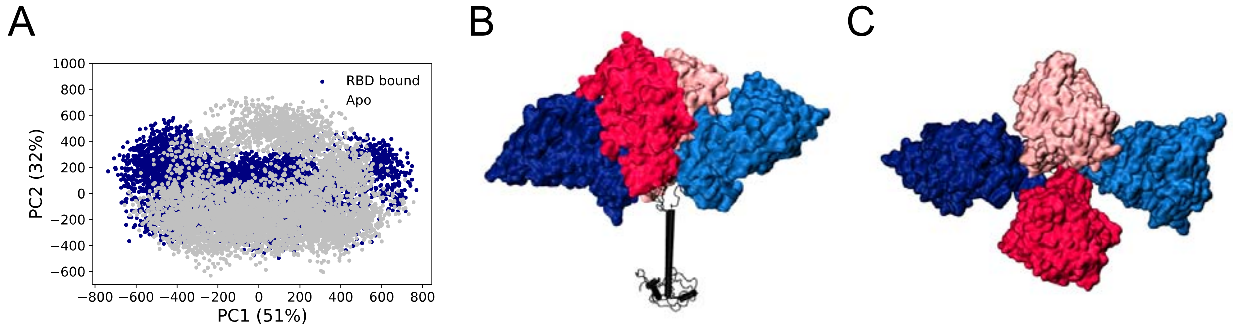

Supplementary Figure 4. (a) PCA plot showing PC1 and PC2 distribution of the apo (grey) and RBD-bound (navy) head coordinates in the simulations. **(b)** Side view and **(c)** top view of the conformations corresponding to the RBD-bound PC1 (in dark and light blue) and PC2 (in dark and light pink) extreme values. Head domains shown in surface representation, with the aligned transmembrane domains in black cartoon.

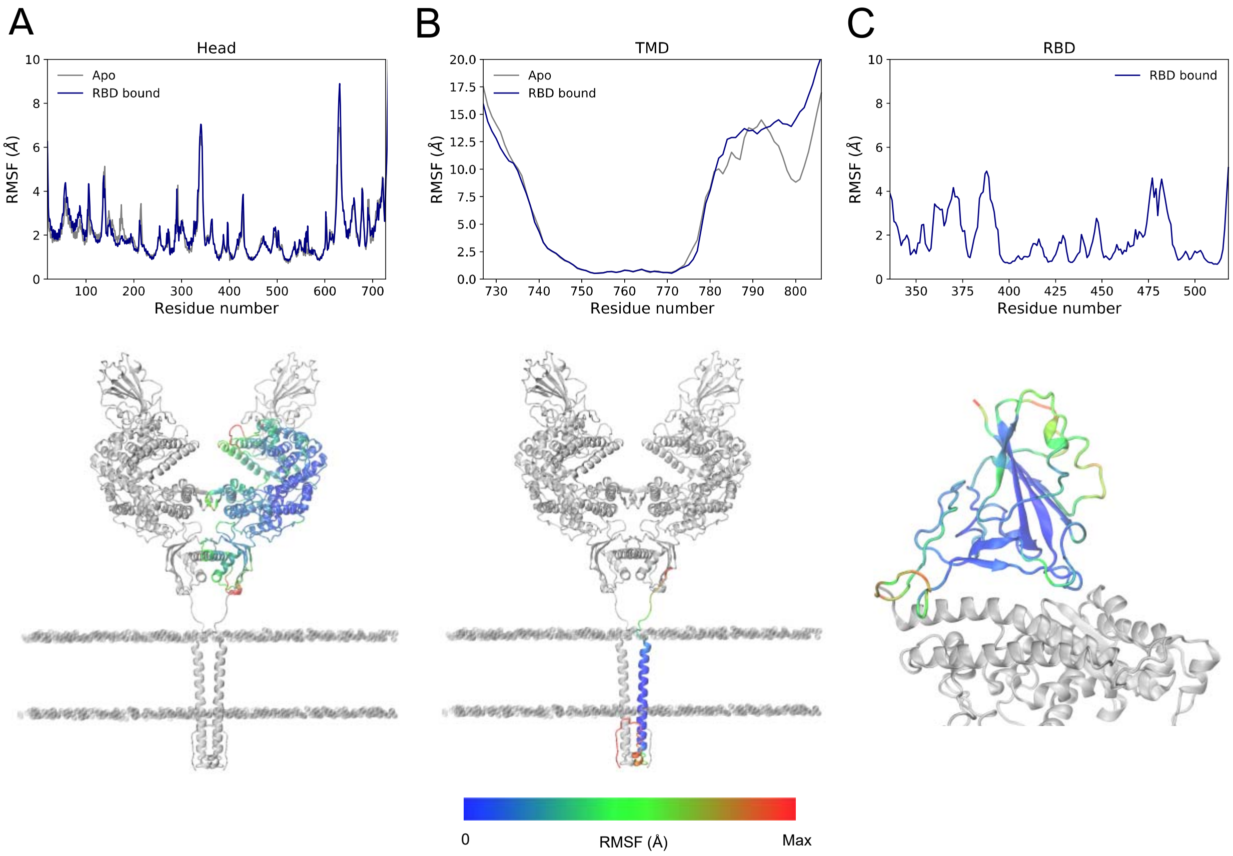

Supplementary Figure 5. Root-mean square fluctuations of backbone atoms for **(a)** ACE2's head, **(b)** ACE2's trans-membrane domain and **(c)** spike's RBD. Quantitative results from the accumulated apo (grey) and RBD-bound (navy) simulations shown at the graphs at the top, with the projection of the RMSF values on the protein structure, colored according to each domain's range of values, shown at the bottom (blue low RMSF, red high RMSF). The other regions of the complex structure are shown in grey for reference.

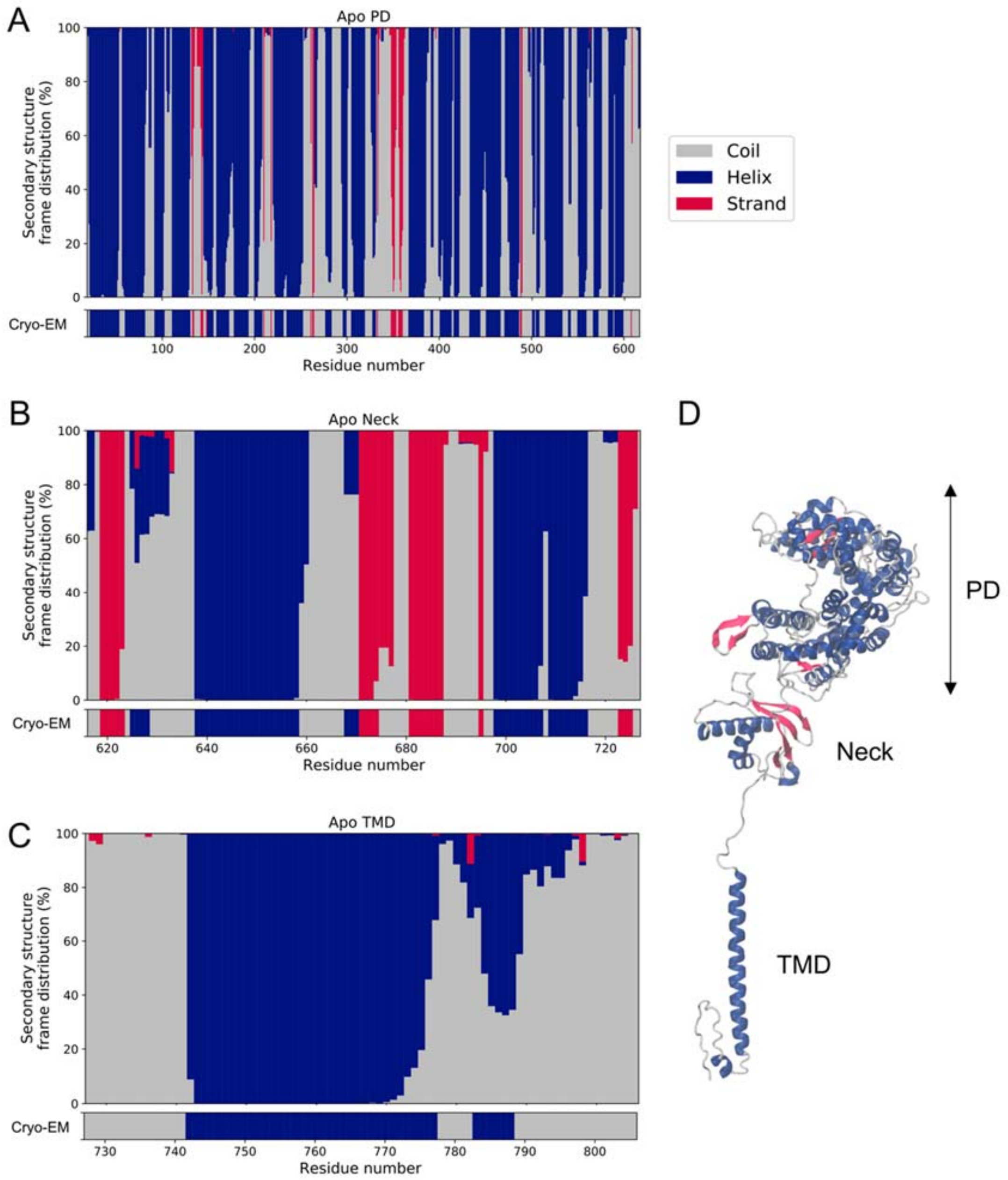

Supplementary Figure 6. Apo simulations secondary structure analysis for **(a)** peptidase (PD), **(b)** neck and **(c)** transmembrane (TMD) domains. The top, large graphs in each panel represent the frame distribution of the secondary structure assignments for each protein residue, colored according to the structure assignment: coil (grey), helix (navy) and strand (magenta). The bottom graph indicates the secondary structure assignment of the reference cryo-EM structure. **(d)** Visual representation of the secondary structure motifs in the initial cryo-EM structure, colored according to the same color scheme.

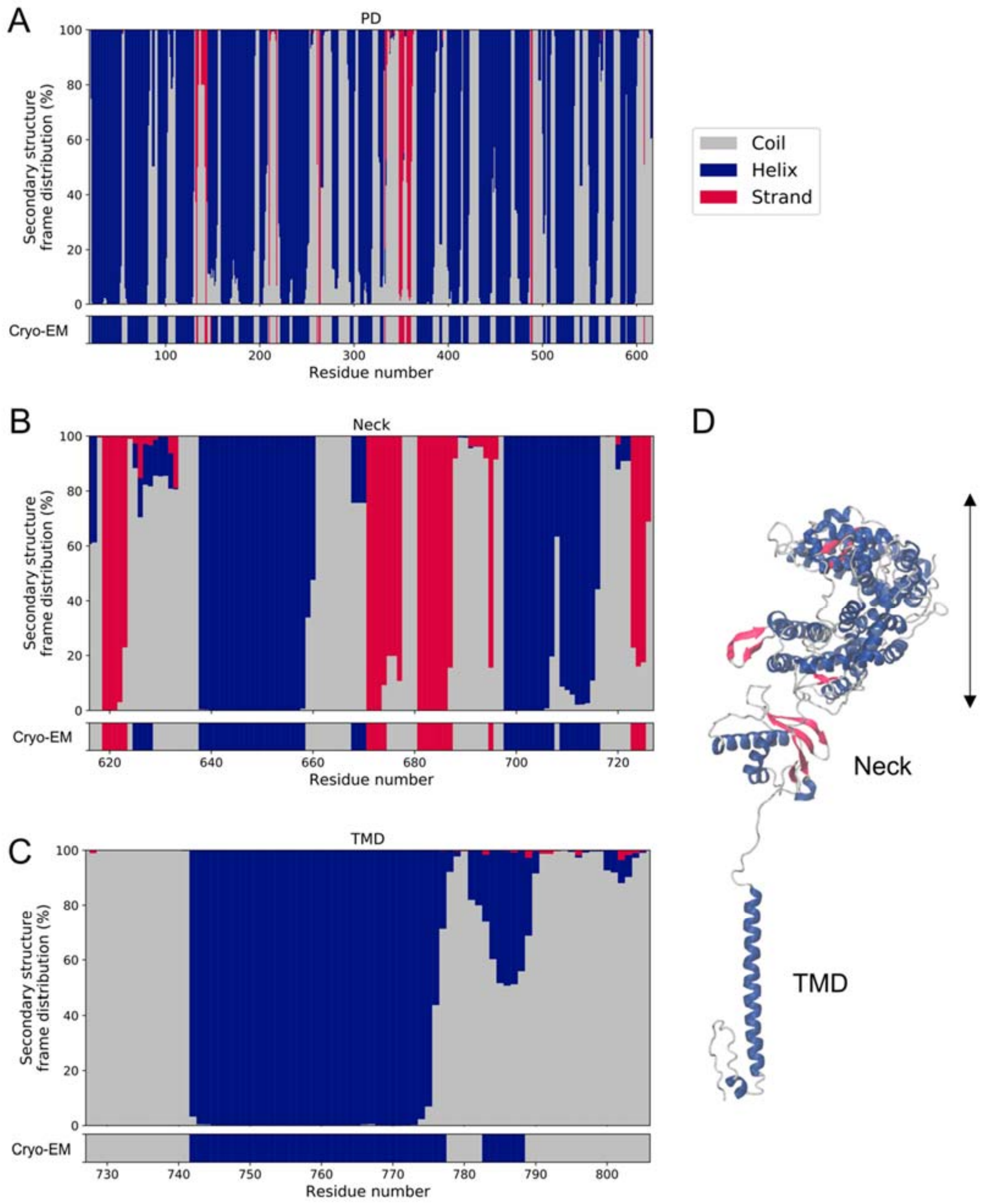

Supplementary Figure 7. RBD-bound simulations secondary structure analysis for **(a)** peptidase (PD), **(b)** neck and **(c)** transmembrane (TMD) domains. The top, large graphs in each panel represent the frame distribution of the secondary structure assignments for each protein residue, colored according to the structure assignment: coil (grey), helix (navy) and strand (magenta). The bottom graph indicates the secondary structure assignment of the reference cryo-EM structure. (d) Visual representation of the secondary structure motifs in the initial cryo-EM structure, colored according to the same color scheme.

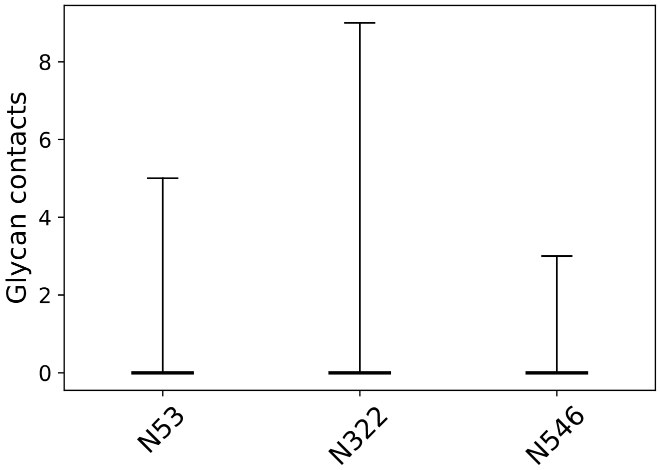

Supplementary Figure 8. N343-ACE2 glycan contacts. Horizontal black lines indicate mean value and whiskers show the total range of the data.

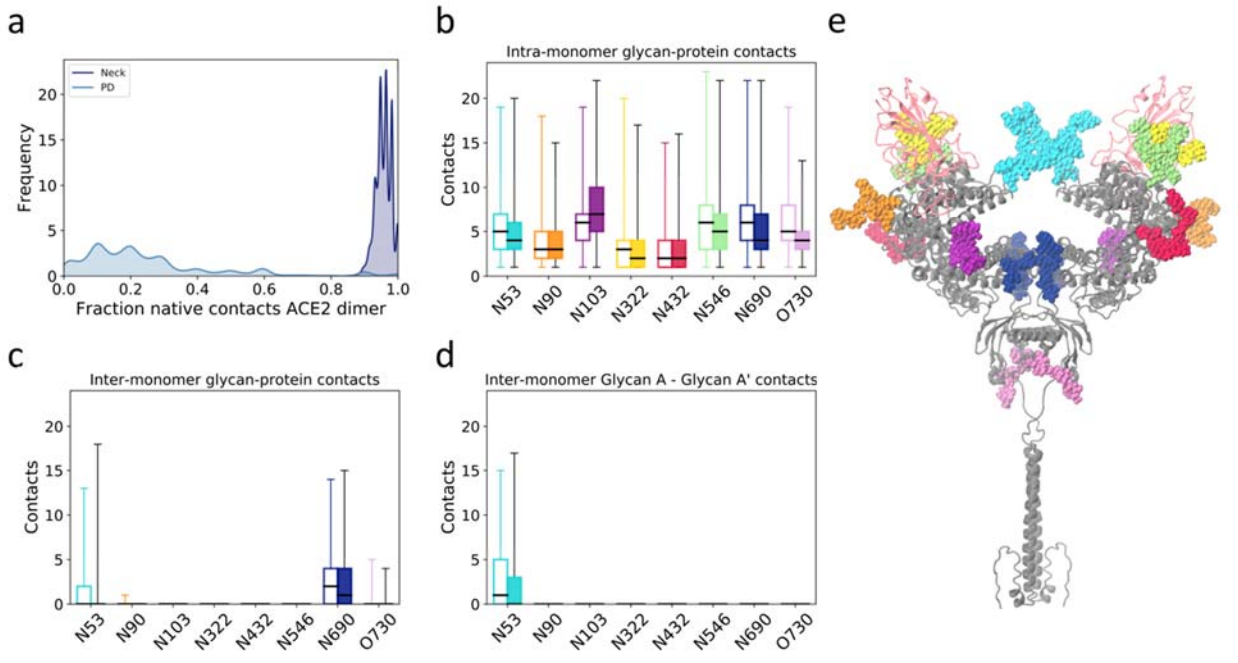

Supplementary Figure 9. Comparison of apo and RBD-bound glycan contacts. **(a)** Fraction of native contacts between ACE2 monomers for the apo simulations, considering only protein components of the glycoprotein. Neck and peptidase domain (PD) interacting regions are indicated separately. **(b)** Total glycan-protein interactions formed within each ACE2 monomer. Results from apo simulations shown in white boxes with colored contour and whiskers, RBD-bound simulations in colored boxes with black whiskers. Horizontal black lines indicate mean value, boxes extend to the lower and upper quartiles, and whiskers show the total range of the data. **(c)** Glycan-protein contacts between glycans in one of the monomer and protein residues from the opposite monomer. **(d)** Glycan-glycan contacts between glycan in one of the monomers and its copy in the opposite monomer (glycan A'). **(e)** ACE2 dimer with glycans in van der Waals representation colored according to figures b-d. ACE2 protein dimer colored grey and RBDs in light pink.

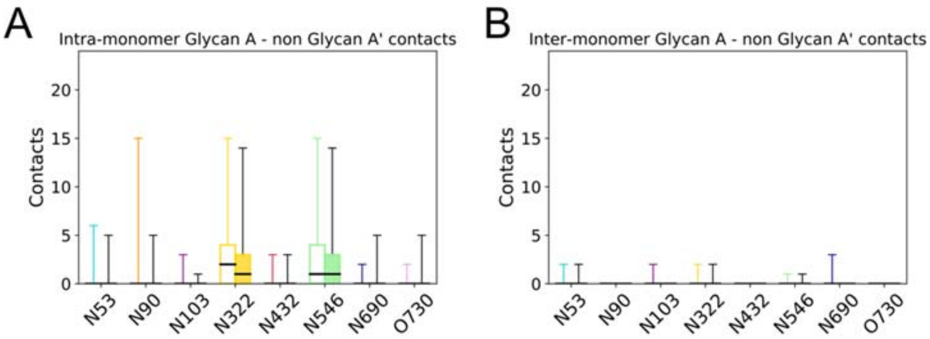

Supplementary Figure 10. ACE2 glycan-glycan contacts from apo and RBD-bound simulations. **(a)** Intra-monomer glycan-glycan contacts. **(b)** Inter-monomer glycan-glycan contacts, excluding contacts between equivalent glycans in the dimer. Results from apo simulations shown in white boxes with colored contour and whiskers, RBD-bound simulations in colored boxes with black whiskers. Horizontal black lines indicate mean value, boxes extend to the lower and upper quartiles, and whiskers show the total range of the data.

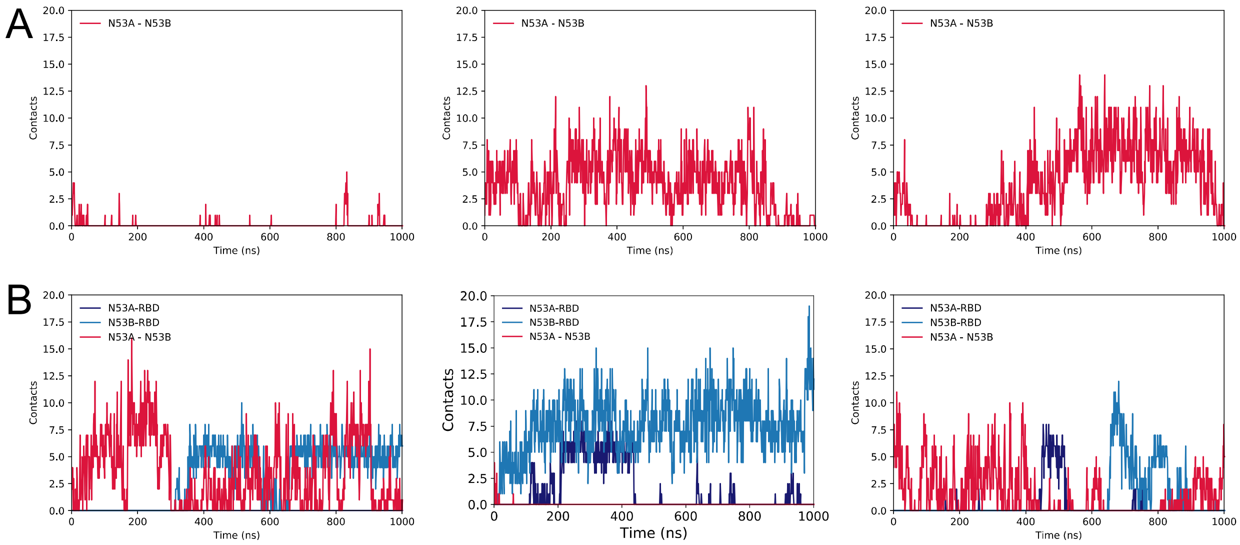

Supplementary Figure 11. Time evolution of N53 contacts. **(a)** N53A-N53B contacts in the homodimer during each replica of the apo ACE2 simulations. **(b)** N53-RBD contacts in each of the heterodimers as well as N53A-N53B contacts in the homodimer during each replica of the RBD-bound simulations.

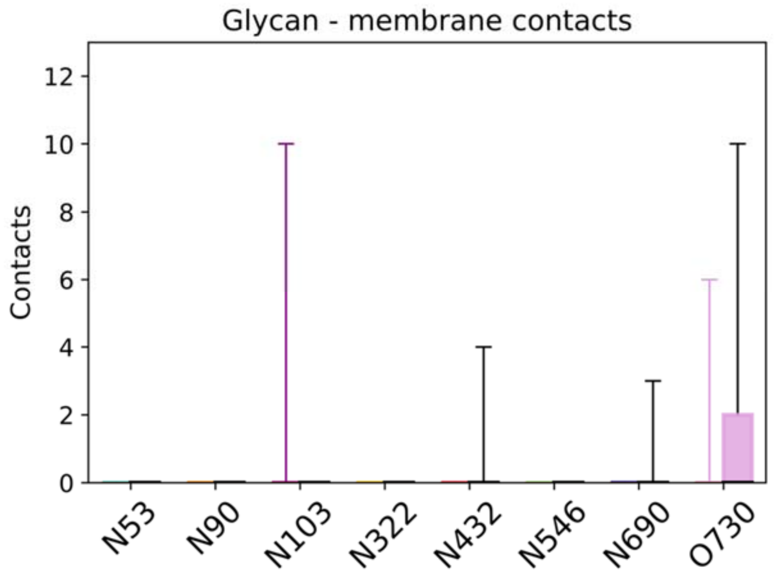

Supplementary Figure 12. Glycan-membrane contacts for apo and RBD-bound simulations. Results from apo simulations shown in white boxes with colored contour and whiskers, RBD-bound simulations in colored boxes with black whiskers. Horizontal black lines indicate mean value, boxes extend to the lower and upper quartiles, and whiskers show the total range of the data.

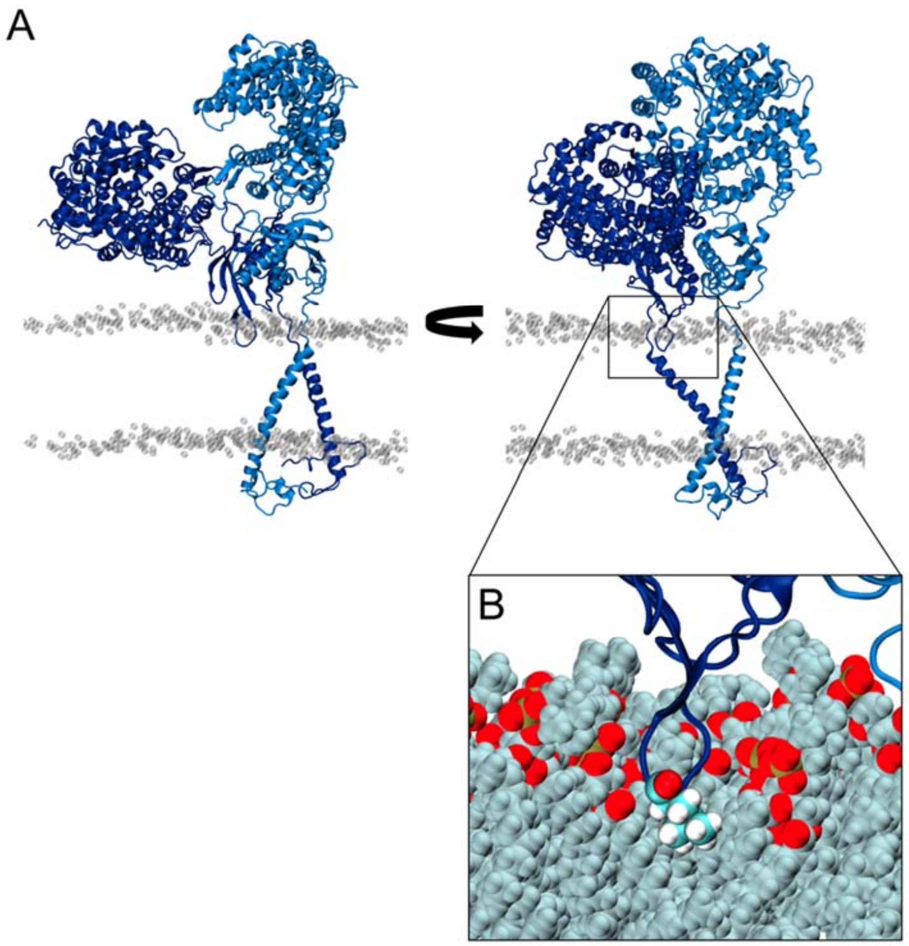

Supplementary Figure 13. (a) Two views of a tilted ACE2 conformation, highlighting the insertion of the neck loop in the membrane. ACE2 monomers colored dark and light blue, and phosphorus atoms from membrane's lipid heads shown in grey, transparent van der Waals representation. (b) Detail of the inserted loop, showing Leu628 (carbon atoms in cyan, hydrogen atoms in white and oxygen atoms in red), and membrane lipids (oxygens in red, phosphorous atoms in ochre and tails in light cyan) in van der Waals representation.
